# Population coding of strategic variables during foraging in freely moving macaques

**DOI:** 10.1038/s41593-024-01575-w

**Published:** 2024-03-05

**Authors:** Neda Shahidi, Melissa Franch, Arun Parajuli, Paul Schrater, Anthony Wright, Xaq Pitkow, Valentin Dragoi

**Affiliations:** 1 Department of Neurobiology and Anatomy, McGovern Medical School, University of Texas, Houston, Houston, TX USA; 2 Georg-Elias-Müller-Institute for Psychology, Georg August-Universität, Göttingen, Germany; 3 Cognitive Neuroscience Laboratory, German Primate Center, Göttingen, Germany; 4 Department of Computer Science, University of Minnesota, Minneapolis, MN USA; 5 Department of Psychology, University of Minnesota, Minneapolis, MN USA; 6 Department of Neuroscience, Baylor College of Medicine, Houston, TX USA; 7 Department of Electrical and Computer Engineering, Rice University, Houston, TX USA; 8 Center for Neuroscience and Artificial Intelligence, Baylor College of Medicine, Houston, TX USA; 9 Neuroscience Institute, Carnegie Mellon University, Pittsburgh, PA USA; 10 Department of Machine Learning, Carnegie Mellon University, Pittsburgh, PA USA; 11 Present Address: Neuroengineering Initiative, Rice University, Houston, TX USA

**Keywords:** Neural circuits, Computational neuroscience, Decision, Reward

## Abstract

Until now, it has been difficult to examine the neural bases of foraging in naturalistic environments because previous approaches have relied on restrained animals performing trial-based foraging tasks. Here we allowed unrestrained monkeys to freely interact with concurrent reward options while we wirelessly recorded population activity in the dorsolateral prefrontal cortex. The animals decided when and where to forage based on whether their prediction of reward was fulfilled or violated. This prediction was not solely based on a history of reward delivery, but also on the understanding that waiting longer improves the chance of reward. The task variables were continuously represented in a subspace of the high-dimensional population activity, and this compressed representation predicted the animal’s subsequent choices better than the true task variables and as well as the raw neural activity. Our results indicate that monkeys’ foraging strategies are based on a cortical model of reward dynamics as animals freely explore their environment.

## Abstract (web summary)

This study investigates self-paced actions in freely foraging macaques. Findings highlight continuously evolving neural components that capture beliefs about latent reward dynamics, which are crucial for informed decision-making in a natural setting.

## Main

While foraging, animals must continuously make decisions about where to search for food and when to move between possible food sources. To survive in environments with sparse resources, animals forage more effectively if they can predict future outcomes before they execute costly actions such as relocation^1–3^. Two major limitations of past neuroscience studies of foraging have impeded our understanding of this natural behavior. First, trial-based tasks are unable to expose the continuous decision-making process during food search and selection, and second, restraining body movements may substantially distort prediction of outcomes of dynamic food sources, as the perception of time is tightly linked to freedom of movement^4,5^ and cortical dynamics^6^.

Trial-based tasks revealed that animals use reward history to detect changes in reward rates^7,8^. We often quantify how an animal adapts to these reward rates by tracking how often it chooses each available option. For example, classical foraging theories revolve around the idea of the matching law^9^: animals dedicate time or effort to an option in proportion to its value according to its reward history. However, a neglected aspect of adaptive behavior is that the animals adjust their response rate, meaning that they choose ‘when’ to forage in addition to ‘where’ to forage. Choosing the response rate systematically is particularly efficient when the time of choice predicts the chance of receiving a reward, that is, in nature as well as in many foraging studies^7–13^. For a restrained animal engaged in a trial-based foraging task, ‘when’ to choose is distorted by the trial structure, while ‘where’ to choose is distorted by a confined spatial distribution of reward. Additionally, examining foraging in trial-based tasks makes it difficult to examine the neural bases of the continuous decisions the animal would make freely about when and where to engage with the task.

The second major limitation of past studies is that experimental restraints used when recording neural activity can distort animal behavior^14–16^. The consequences of physical restraints may be especially dramatic on food-seeking behavior because animals use head and body movements to gather information from their environment for foraging^17,18^. Furthermore, the cortical activity differs when the animals aim for targets that are far from their immediate reach^19^. The restraints may also affect when the animals choose to act, particularly for trial-free experiments for which timing is crucial.

In this Article, to circumvent these limitations, we designed a trial-free task where animals can forage freely, without bodily restraints. We examined how dynamic task variables influence rewards and neural activity, and subsequently, how this activity influences foraging behavior. Animals were allowed to continuously interact with the task and explore a wide range of reward expectancies by choosing when and where to act. We found that animals adjust their foraging on the basis of deviations from theoretical reward predictions, reflecting subjective reward expectations and leading us to two hypotheses about brain computations. First, the subjective estimates of reward predictors should be decodable from the brain. Second, animals should choose the time and the place of foraging attempts according to these neural estimates. We tested these hypotheses by recording from neurons in the dorsolateral prefrontal cortex (dlPFC), an area where neural activities encode reward^13,20,21^ and are related to memory^22^ and action preparation^23,24^. Here, we show that single neurons had mixed selectivity^25^ to experimental variables and, conversely, those variables were distributed across many neurons. Finally, this distributed representation accurately predicts where and when an animal will forage next.

## Results

**Fig. 1:**
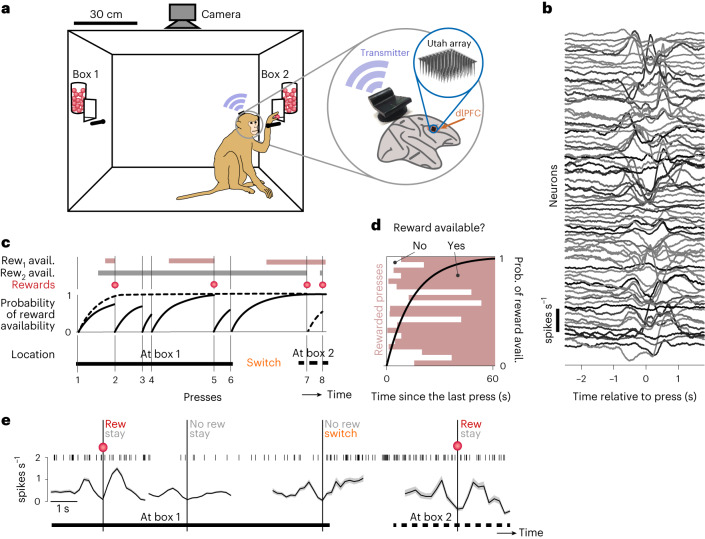
Foraging in freely moving monkeys while population activity in the prefrontal cortex is recorded wirelessly. **a**, Left: schematic of experimental setup with two reward boxes, two buttons and an overhead camera. Right: the location of the Utah array in the dlPFC (area 46) and wireless transmitter. **b**, Press-averaged firing rates of 80 single and multi-units recorded simultaneously. **c**, An illustration of task dynamics with eight hypothetical presses (vertical lines) in the concurrent variable-interval foraging task. In this illustration, the monkey responds six times on box 1, then switches to box 2 and responds twice. Therefore, press 6 is considered a press with a switch choice. The first two rows show the independent telegraph processes determining the reward (rew.) availability at boxes 1 and 2. In the example shown, press numbers 2, 5, 7 and 8 were rewarded (third row, red). The time dependence of the probability of reward availability is shown in the fourth row (see **d** for a different representation). **d**, An alternative illustration to clarify the relationship between the probability (prob.) of reward availability (avail.) and the waiting time. The shaded area shows when a reward is available after each of the 20 presses on box 1 (some of them shown in **c**). The black trace associated with the *y* axis on the right shows the probability of reward availability (Methods and Extended Data Fig. 2a), which at each time is in fact the proportion of pink bars, out of 20 trials with pink bars. **e**, The spike train of one example neuron on the timescale of four consecutive presses showing a variety of events (top row). Event-locked average firing rates of the same neuron are shown in the bottom row for conditions with reward/no reward and stay/switch choices. For ease of visualization, this example used a neuron with a relatively low firing rate compared to others in the population (compared with **b**).

**Extended Data Fig. 1:**
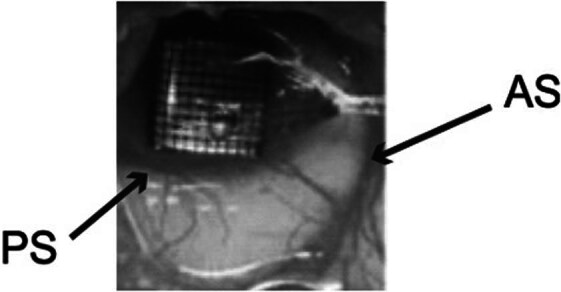
The MEA was implanted in the dorsolateral prefrontal cortex. The location of a 96-channel Utah array in dlPFC (area 46) on the left hemisphere of monkey G. The arcuate sulcus (AS) and principal sulcus (PS) are marked.

Monkeys (*n* = 2) were exposed to two concurrent reward sources on a variable interval (VI) schedule^10^. We made it costly for the animal to switch between reward sources by placing them 120 cm apart (Fig. 1a, left). Animals freely interacted with the task equipment, and we did not impose a trial structure or a narrow response window (Methods). A multi-electrode Utah array was chronically implanted in the dlPFC (Extended Data Fig. 1), and measured spiking activity was collected using a lightweight, energy-efficient wireless device (Fig. 1a, right and Fig. 1b)^26^. The experimental setup was designed for the effective transmission of a low-power electromagnetic signal (Methods)^27,28^.

**Extended Data Fig. 2:**
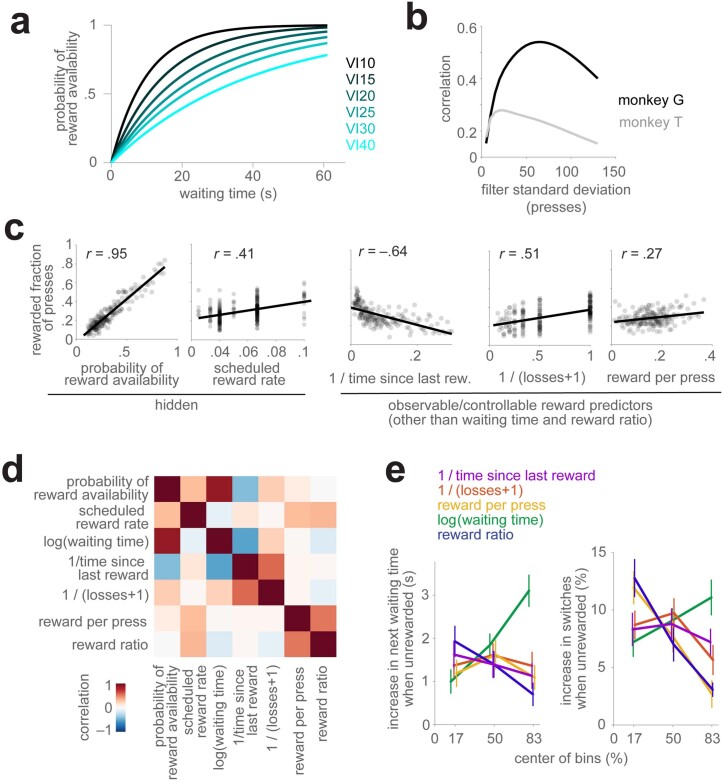
A comprehensive set of behavioral and experimental variables was examined before selecting the reward predictors. **(a)** The probability of reward availability as a function of the scheduled reward rate and the time since the preceding response on the same box. **(b)** The Pearson correlation coefficient between the scheduled and the (observable) reward ratios is calculated using a recent sequence of reward outcomes defined in the main text. The recency was imposed by choosing the standard deviation of a causal half-Gaussian filter (x-axis). For each monkey, the reward ratio was calculated using the standard deviation for achieving the maximum correlation with the scheduled reward ratio. **(c-e)** Other observable/controllable variables that the animal might have used to predict rewards. **(c)** Similar to Fig. 2a, but for the inverse of the time since the last reward, the inverse of the number of unrewarded presses (losses), and the reward per press (the binary sequence of rewarded (1) and unrewarded (0) presses, filtered using the same causal half-Gaussian filter that was used for calculation of the reward ratio). **(d)** Correlation matrix between observable and unobservable reward predictors. Based on this matrix, the waiting time was chosen as one reward predictor because it was maximally correlated with the hidden probability of reward availability, and the reward ratio was selected as another reward predictor because it was maximally correlated with the scheduled reward rate and minimally correlated with the waiting time. Other predictors were omitted because they were correlated with either of these two variables (|r | >0.21). **(e)** Change of the next action (n = 8862) as a function of each reward predictor candidate that was discretized into three bins with an equal number of presses in each bin. The x-axis shows the center of the bins. The y-axis shows the excess percentage of switches or waiting time when unrewarded compared to when rewarded. Two chosen reward predictors, waiting time and reward ratio, linearly predict the change of the following action. Data are presented as mean values +/- SEM.

Rewards on both sides (box 1 and box 2) became available at exponentially distributed random times after the animal obtained a previous reward. The reward availability was hidden from the monkey. Once becoming available, each reward remained available until the animal pressed a button, at which time the reward was delivered (Fig. 1c). The distribution of waiting times before a reward became available could have different mean times or ‘schedules’ for each side (that is, constant hazard rates; Fig. 1c). Schedules were chosen from 10, 15, 25 or 30 s and were constant for a block of rewards. Multiple schedules allowed us to diversify the response dynamics of the animals^10^. Each experimental session contained two to four blocks with 34 or 66 rewards in each block. Given the constant hazard rate and the fact that rewards never disappeared once available, the probability of reward availability increased exponentially toward 1 with the time elapsed since the last press (the waiting time), with a time constant given by the reward schedule (Methods, Fig. 1d and Extended Data Fig. 2). Since the monkey chose when to respond, its decisions influenced the probability of reward availability (Fig. 1c). An ideal observer that did not know the schedule or availability should track the time and reward histories, so we hypothesize that animals attempt to maximize their reward by tracking these quantities, referred to as the reward predictors, to determine when and where to respond.

We examined whether the firing rates of neurons in dlPFC represent the reward predictors as they are continuously evolving in time (monkey G: 11 sessions and monkey T: 19 sessions; *n* = 1,323 single and multi-units). Additionally, we extracted the neurons’ press-locked events, that is, firing rates a few seconds before and after each press (Fig. 1b). The continuous-time neural activity allowed us to understand how continuous representations of task variables in dlPFC leads to the animal’s choice to press. The press-locked neural activity explained how the state of these representation, before a press, combined with the new information, which is the reward outcome, predict where and when the animals press next. The continuous spike raster and the press-locked firing rate of a sample neuron (Fig. 1e) are shown for four consecutive box presses with different reward/choice outcomes: an unrewarded press followed by a switch to the other box, an unrewarded press when the animal stayed at the same box and two rewarded presses when the animal stayed at the same box. The fourth outcome, switching to the other box after a rewarded press, accounted for only 2% of the presses, so we do not show it in this example.

Here we explain how we identified reward predictors, variables that the animal can either observe or control and that they potentially use to estimate the chances of rewards. Consequently, we determined whether these variables empirically predicted the next reward outcome in our experiment. As the stochastic rewards do not always match the prediction, we examined the consequences of prediction violations on animals’ choice of box and time of press. Next, we used canonical correlation analysis (CCA) to identify the neural representation of these variables in the population of recorded neurons in dlPFC. Finally, we tested whether these representations predicted the animal’s choices in advance.

### Predictors of the next reward

According to the marginal value theorem of foraging theory^1^, an animal could optimize its reward while minimizing travel costs by estimating the box schedules, tracking the temporal evolution of the probability of reward availability and using them to choose when and where to search for reward. Although the probability of the reward availability is the best predictor of the randomly generated reward, it was completely unobservable to the animals in our experiment. However, other predictive variables were observable or controllable by the animals, such as the waiting time between the presses or the reward ratio, defined as the proportion of the current option’s recently delivered reward compared to all recently delivered rewards from either box. The recent history was defined by applying a causal half-Gaussian filter to the binary sequence of delivered (1) or denied (0) rewards^7,8^. The waiting time, together with the scheduled reward rate, determines the probability of reward availability (Methods and Extended Data Fig. 2a). The reward ratio, when tracked on a timescale relevant to the volatility of the environment^7^, is a proxy for the scheduled reward ratio, defined as the ratio of the scheduled reward rate on the current box and the sum of the scheduled reward rates of two boxes.

**Fig. 2:**
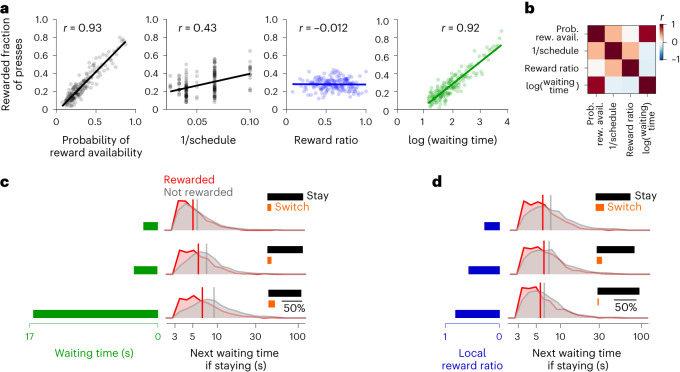
Reward predictors, together with the reward outcome, determine the choices and the next waiting time. **a**, The predictability of the next reward from experimental and behavioral variables: 8,862 presses from 30 sessions were pooled together and binned into 50 press bins according to each experimental variable. The rewarded fraction of presses was calculated in each bin, then the Pearson correlation coefficient was calculated across bins between the average of the experimental variable and the rewarded fraction of presses. **b**, A correlation matrix of the task variables in **a**. **c**, Histograms of the next waiting time for rewarded and unrewarded presses that were made after a short, medium or long wait, determined by equal intervals in the percentile of the presses. Inset: an increase in the probability of switching when not rewarded after a short, medium or long wait. The probability of switching after being rewarded was less than 2% and therefore excluded from this analysis. **d**, The same as **c**, but for reward ratio instead of waiting time.

As the scheduled reward ratio changes without warning from block to block, we maximized the correlation of the scheduled reward ratio with the animal’s observed reward ratio by tuning the width of the causal half-Gaussian filter mentioned above (Extended Data Fig. 2b). We assessed how well each variable predicted the reward by correlating the rewarded fraction of presses with that variable before each press. Specifically, we pooled 8,862 behavioral presses from 30 sessions of two monkeys, binned them according to each hidden or observable/controllable variable so that there were 50 presses in each bin, calculated the fraction of rewarded presses within each bin (Fig. 2a), and computed the Pearson correlation between the binned variable and rewarded fraction of presses. Naturally, the probability of reward availability was highly correlated (*r* = 0.93; Fig. 2a) with the rewarded fraction of presses. The scheduled reward rate was correlated with the fraction of rewarded presses as well (*r* = 0.43; Fig. 2a). This correlation is weaker than the correlation of the waiting time with the fraction of rewarded presses (*r* = 0.92; Fig. 2a) because the probability of reward availability is determined by both waiting time and the scheduled reward rate, and the animals choose a wide range of the waiting times, diluting the prediction of the scheduled reward rate alone.

Although the waiting time was highly predictive of the next reward, the reward ratio potentially plays an important role in animal’s subjective reward expectation^8^. The reward ratio was not correlated with the fraction of rewarded presses (Fig. 2a, *r* = −0.012). However, it was positively correlated with the scheduled reward rate on the side that the animal pressed (*r* = 0.32), meaning that it might be used by the animals as an observable estimation of the hidden reward rates. Moreover, it was only weakly correlated with the log of waiting time (*r* = −0.140.14), meaning that it may be considered by the animals as a source of information, independent from the waiting time. We refer to the waiting time and the reward ratio as the reward predictors because they may be used by the animals to predict the reward, and therefore may play a role in determining the animals’ reward expectation (for the analysis of other observable reward predictors, see Extended Data Fig. 2c–e).

### Do reward predictors determine ‘when’ and ‘where’ to press?

**Extended Data Fig. 3:**
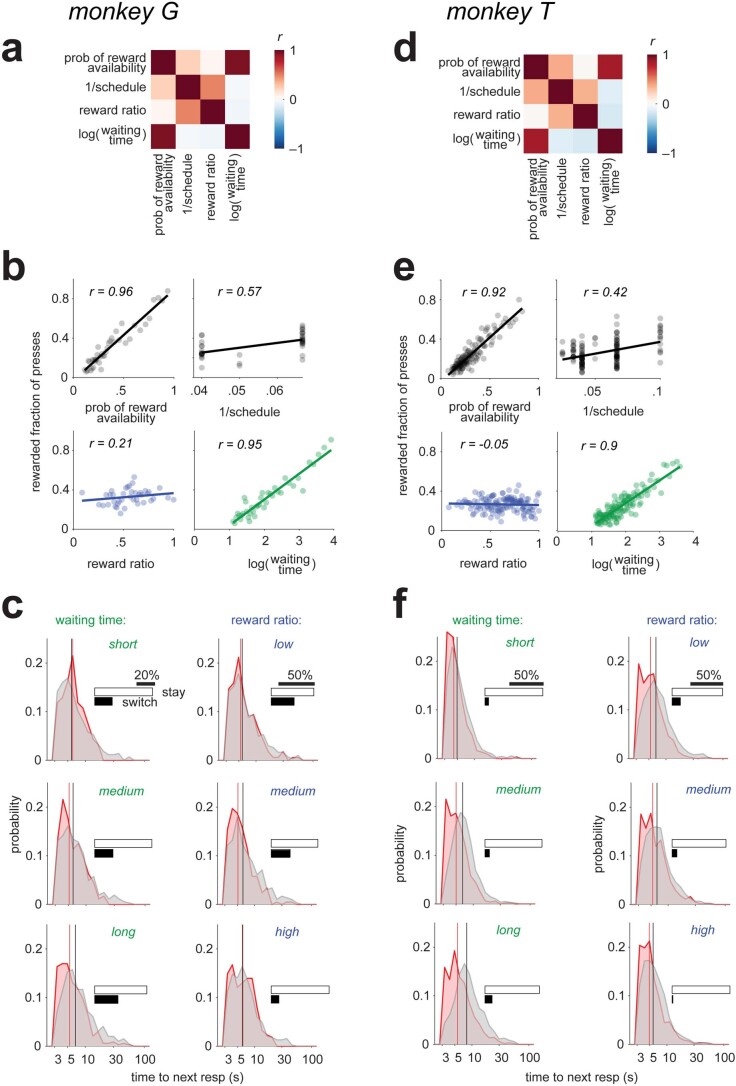
Reward predictors, together with the reward outcome, determine the choices and the next waiting time. Same as Fig. 2, but separated for monkey G and T. (**a**,**d**) Correlation matrices of the task variables. (**b**,**e**) Predictability of the next reward from experimental and behavioral variables. The rewarded fraction of presses was calculated in each 50 press bin, then the Pearson correlation coefficient was calculated across bins between the average of the experimental variable and the rewarded fraction of presses. **(c,f)** Left: histograms of the next waiting time for rewarded and unrewarded presses that were made after a short, medium or long wait, determined by equal intervals in the percentile of the presses. Inset: an increase in the probability of switching when not rewarded after a short, medium or long wait. The probability of switching after being rewarded was less than 2% and therefore excluded from this analysis. Right: the same as left, but for reward ratio instead of waiting time.

Although the subjective reward expectation is not directly measurable, we might infer changes in the animals’ reward expectation from the animals’ next choice, after a reward is delivered or denied. For example, an animal may realize that waiting longer increases its chances of receiving a reward, so we expect that an unrewarded press after a long wait might lead it to wait even longer between presses at the current box. Alternatively, the animal may realize that the waiting time for getting a reward at the current box is too long. Therefore, it may switch to the other box anticipating a better reward rate. We thus hypothesized that the animals’ decision on where and when to press depends upon the reward predictors, as the basis of animals’ reward expectation. We evaluated this hypothesis by analyzing the effect of such reward predictors on the probability distribution of the next waiting time and the probability of switching. These events were grouped depending on whether presses were rewarded and occurred after a short (3–5 s), medium (5–8 s) or long (8–60 s) wait (Fig. 2c and Extended Data Fig. 3, separated for monkeys). An unrewarded press increased the next wait by 10% (area under the receiver operating characteristic curve (AUC) of 0.53 ± 0.03) after a short wait, by 28% (AUC of 0.59 ± 0.02) after a medium wait and by 42% (AUC of 0.59 ± 0.02) after a long wait, each compared with the corresponding average waiting times for rewarded presses. Moreover, the probability of switching to the other box increased with the duration of unrewarded waits (9.5%, 10.2% and 16.5% more switches after a short, medium and long waiting time; Fig. 2c, insets). These choice differences (to continue pressing the button for the same box or switch to the other box) and the next waiting time when choosing to press on the same box, demonstrate that animals base their expectation of reward on their waiting time and adjust their behavior by waiting longer before the next press or switching to the other box when this expectation is not met. While previous studies point to melioration, that is, following the current flow of reward delivery^9^, we provide evidence of more temporally structured computations: the animals predict the chance of the next reward as they choose how long to wait before making the next press and adjust the waiting time when their expectation is not met. A key to this finding was a trial-free task, allowing animals to experience a wide range of waiting times and spontaneously discovering that longer intervals yielded a higher chance of receiving a reward.

The animals might also develop expectations about the quality of the current box from the reward ratio. Again, we can infer these expectations indirectly through changes in the next waiting time and choices. After unrewarded presses, animals waited longer and switched more, with the smallest changes for biggest reward ratios (Fig. 2d; 23%, 18% and 15% longer unrewarded waits and 19%, 12% and 3% switches after a low, medium and high reward ratio, respectively). This suggests that animals require stronger evidence to override a better reward history.

Altogether, this provides evidence that an animal’s policy on when and where to press depends on whether the box delivers a reward, as expected after a long waiting time or a high reward ratio. We inferred that animals update their expectation when those expectations are violated by the lack of an expected reward. This policy is a case of ‘learning a guess from a guess’^29^, which is useful in the absence of sensory evidence directly cueing the probability or availability of reward. To provide further evidence that the waiting time and reward ratio underlie animals’ reward expectation, we examined their encoding in the recorded neural population.

### Task-relevant activity in dlPFC

**Fig. 3:**
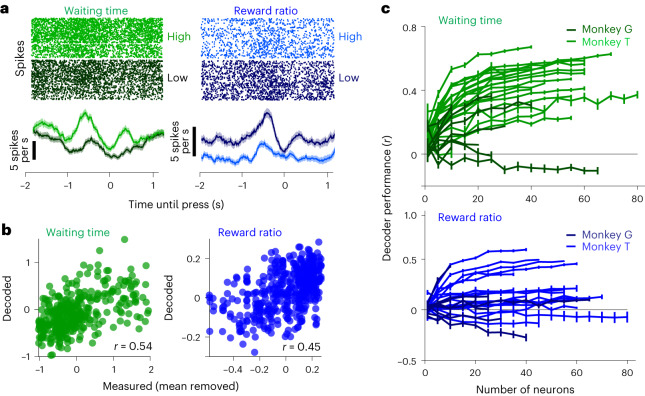
Neuronal populations encode variables of the reward dynamics. **a**, Sample neurons for which the pre-press firing rate covaries with either waiting time (left) or reward ratio (right). The firing rate was calculated for a 200 ms sliding window starting 2 s before and ending 1 s after presses. Firing rates were averaged across presses with low (<20th percentile, gray) and high (>80th percentile, colored) of either the waiting time or the reward rate. Data are presented as mean values ± s.e.m. **b**, Decoded and measured waiting time (left) and reward ratio (right) for two sample sessions. Forty-five neurons in the session on the left and 60 neurons in the session on the right were used. The shown value of Pearson correlation coefficient is the average value across the cross-validated sets in each session. **c**, Decoding the waiting time (top) or the reward ratio (bottom) for 30 sessions as a function of the number of neurons used as predictors. The predictor neurons were chosen randomly from the population. Data are presented as mean values ± s.e.m. across 20 randomly selected subset of units. Sessions of monkey G are shown with a darker color, and sessions of monkey T are shown with a lighter color.

Before a motor action, the activity of neurons in the dlPFC is correlated with the value of a visually cued expected reward^20^ or the probability of reward, estimated by the recent history of reward delivery^13^. Therefore, we hypothesized that the activity of dlPFC neurons, before each press, encodes the reward expectation for that press, for the range of the reward predictors variables observed or generated by each animal. For example, the neuron in Fig. 3a, left, activates more before a press following a long wait (top 20% of waiting times in that session) compared with a short waiting time (bottom 20%; Wilcoxon rank-sum test, $P$ ≪ ${10}^{-3}$). Similarly, the neuron in Fig. 3a, right, activates more when the reward ratio before a press is in the bottom 20% compared with when it was in the top 20% (Wilcoxon rank-sum test, *P* ≪ 10^−3^).

**Extended Data Fig. 4:**
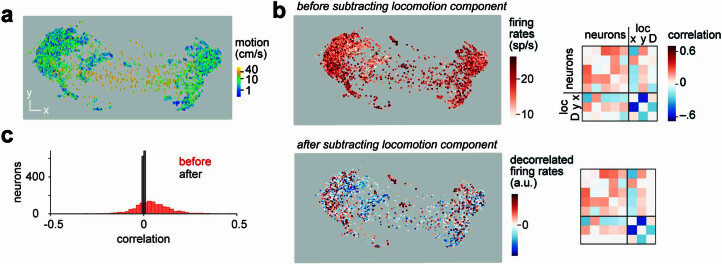
The neural activities were orthogonalized to monkeys’ locomotion before Figs. 3–5 analyses. **(a)** Monkey locomotion in a sample session for the press-locked time bins (< 3 s before or > 1 s after a press). Each dot represents the animal’s position in space, sampled every 200 ms. **(b)** *Left*: Population-averaged firing rates for each time bin in (a). *Top* and *bottom* show firing rates before and after subtracting the vector projection of locomotion. *Right:* Matrix of correlation coefficients between pre-press firing rate of each neuron and between the monkey’s locations Loc X and Loc Y and locomotion Loc D. For clarity, only an arbitrary subset of neurons is shown. **(c)** Histogram of correlation coefficients computed as in (b), but for all recorded neurons.

**Extended Data Fig. 5:**
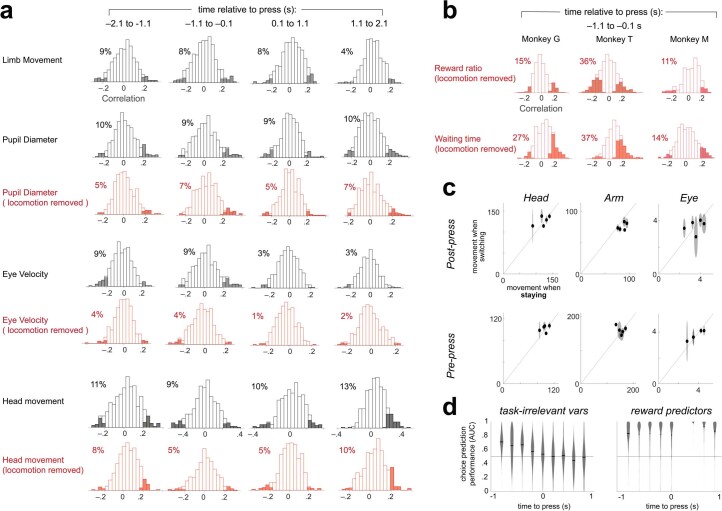
Effect of eye and limb movements on the neural activities and choices. **(a)** Histograms of correlation coefficients between the limb and eye movements and individual neuron activities. Bars are shaded for statistically significant correlations, and the inset indicates the percentage of significantly correlated neurons. A deep artificial neural network was trained using DeepLabCut (Mathis et al., 2018) to localize monkey M’s shoulder, elbow, and paw of the forelimb contralateral to the recording site (right limb) in each overhead video frame of the foraging animal. We could compute average limb movement in any desired time interval using those three limb markers. We considered time intervals around button presses (–2 s, +2 s) and computed the average limb speed and firing rate of all the dlPFC neurons of monkey M in non-overlapping time-bins of 200 ms width. The pupil diameter and the eye velocity were computed with the same method as in ref. ^26^ **(b)** For comparison, the correlation coefficient of the reward predictors with firing rates of dlPFC neurons for monkeys G, T, and M **(c)** Magnitude of movements of the head, arm, and eye are similar whether the animal chooses to stay (horizontal axes, arbitrary units; 5 sessions, 238–336 presses per session) or switch locations (vertical axes, arbitrary units; 5 sessions, 3–27 presses per session) around the time of a button press. Points depict mean movement magnitudes, and widths of shaded ellipses indicate standard errors of the means along each axis. The top row shows movements after presses, and the bottom row shows movements before presses. **(d)** Prediction of monkey M choices, cross-validated using 1000 sub-samples of presses (80% of presses used for training, 20% for testing). The prediction performance was calculated as the area under the curve of the output of logistic regression (as in Fig. 5c), except that the predictors were the task-irrelevant variables, eye, body, and head movements, and pupil diameter (left) or reward predictors (right). The mean (black line) and the Gaussian-smoothed distribution (gray shades) across 1000 sub-sampled test data are shown.

As task-irrelevant variables such as locomotion, limb and eye movement and pupil size before or after presses may influence dlPFC activity^30^, we performed control experiments to quantify the correlation between task-irrelevant variables and neural activity. First, our control experiments in which animals moved to receive reward from the same boxes as in Fig. 1a revealed that eye movements have only a minor influence on neuronal activity while animals interacted with the box, although they have a stronger influence during locomotion (*r* = 0.16, *t*-test, $P$ ≪ ${10}^{-3}$, for eye velocity, and *r* = 0.13, *t*-test, $P$ ≪ ${10}^{-3}$, for fixation rate^27^). We thus decorrelated the neural activity from the locomotion by projecting neural activity onto the subspace orthogonal to locomotion (Methods) such that the remaining neural activity was uncorrelated with locomotion (Extended Data Fig. 4). Second, one animal performed the same task as presented here, while its arm movements, pupil diameter and eye velocity were monitored using the same eye tracking method as in ref. ^27^. We found ≤9% of neurons in dlPFC with significant (*P* < 0.01) correlation with the arm movement (Extended Data Fig. 5) in 1 s time intervals starting 2 s before and ending 2 s after presses. Pupil diameter was correlated with ≤10% of neurons. However, after we decorrelated the neural activity from the locomotion, the percentage of neurons with a significant correlation with the pupil diameter dropped to ≤7%. Similarly, the percentage of neurons with a significant correlation with the eye velocity dropped from ≤9% to ≤4%. As decorrelating the neural activity from locomotion also decreases the correlation between the neural activity and other task-irrelevant variables, we focused our analysis for the rest of this study on the neural activity that was decorrelated from the locomotion.

### Decoding reward predictors from the neural population

Since the waiting time influences both future behavior and the reward probability when the button is pressed, we examined how the neural activity encodes waiting time just before a button press. We measured the spike counts in a 1 s interval (that is, a ‘pre-press’ interval from −1.1 to −0.1 s) for each neuron (*n* = 1,323 single and multi-units). This time interval was selected since the arm movement starts approximately 0.5 s before the press is recorded, and the modulation of neural activity typically starts around 0.5 s before that movement^31^.The pre-press firing rate of the neuron in Fig. 3a, left, was correlated with the waiting time (Spearman correlation coefficient of 0.24; *t*-test, $P$ ≪ ${10}^{-3}$; Fig. 3b, left). For the entire population of cells, around 35% of neurons exhibited a significant Spearman correlation (*t*-test, *P* < 0.01; 31% positively correlated and 4% negatively correlated; monkey G: 27%, and monkey T: 37%).

To further examine how information about the waiting time is distributed across neurons, we decoded the waiting time from population activity before each press using the spike counts of randomly subsampled sets of neurons (for a description of the regression-based decoder analysis, see Methods). Our decoder analysis revealed that even random neural subpopulations encode the waiting time (Fig. 3c; Wilcoxon rank-sum test with false discovery rate, with multiple comparison correction (WRFDR), *P* ≤ 0.01).

Furthermore, consistent with previous reports^8,13,21,32^, we found that dlPFC neurons encode the reward ratio. Over the entire population, there was a significant correlation between the pre-press firing rate and reward ratio (*t*-test, *P* < 0.01) for 23% of the neurons (9% positively correlated and 14% negatively correlated; monkey G: 12%, and monkey T: 26%). Decoder performance for the reward ratio was higher than chance (WRFDR, *P* < 0.01) when we used a subpopulation of one or more neurons as the predictors. Taken together, these results indicate that both reward predictors are encoded in the pre-press neural activity at the individual neuron and population levels. This finding provides further evidence that the animals’ reward expectation is founded on the chosen reward predictors.

### Identifying continuous task variables in a latent space

**Fig. 4:**
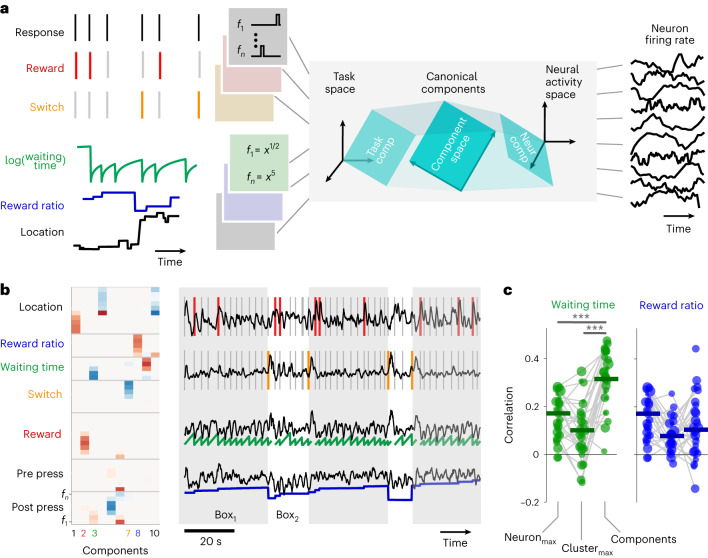
Canonical components of the neural population represent task variables in continuous time. **a**, An illustration of the CCA for finding a reduced-dimensional space in the task space and the corresponding subspace in the neural activity space. The canonical components define the maximally correlated subspaces between the task variables and the neural (neur) activity. The 51-dimensional task space was made from 6 task variables by passing each variable through a set of basis functions (Methods). In brief, the basis functions were pulse-shaped temporal delay filters for press, reward and choice events. For continuously evolving task variables (the waiting time, the reward ratio and two-dimensional location), the basis functions were a set of instantaneous power functions. Overall, 51 predictors were made using these six task variables. The neural space was made using all simultaneously recorded neurons. **b**, Left: the weight of the contribution of each task variable in the first ten canonical components, sorted in the descending order of the correlation between the projection of each component in the task and neural spaces. The indices of the components representing waiting time, reward ratio, reward and choice are color coded for easier association. Right: neural representation of four task variables: reward, choice, waiting time and reward ratio for the same sample session on the left. The component that was associated with each of these four task variables was identified as the component for which the absolute value of the weights was highest, compared with the weights for the other task variables. **c**, Cross-validated Pearson correlation coefficient between the reward predictors, waiting time (left) and reward ratio (right; *P < 0.005), with either individual neurons or clusters of five or more neurons (Extended Data Fig. 6) that are maximally correlated with each reward predictor, compared with the correlation coefficient between the reward predictors and the canonical components (WRFDR; left: $P$ ≪ ${10}^{-3}$ for clusters and 0.003 for neurons; right: *P* > 0.1). Each data point is associated with one session (*n* = 30).

Unlike waiting time, the reward ratio jumps discretely at press times. We aimed to gauge the waiting time’s explanatory power for the continuously evolving neuron activity. We attempted to fit the variability in a neuron population using a weighted sum of task-related variables and basis functions^33–35^. Some of these variables were event based (presses, reward delivery and choice to stay or switch location), while others evolved continuously (waiting time, reward ratio and location within the cage). For event-based task variables, each event raster was filtered with a 200 ms boxcar and then shifted to a variety of offsets^36^ (Fig. 3e). For continuously evolving task variables, we used monomial basis functions with powers of 0.5, 1, 2, 3 and 5 (Fig. 4a). Neural activity was smoothed by a 1 s sliding window. To concentrate our analysis on times when animals were engaged in the task, we excluded time bins preceding or following any presses by more than 5 s.

To identify the latent representation of task variables in the neural space, we used CCA to find components that are shared between the task and the neural spaces. CCA finds these canonical components by applying singular value decomposition to the cross-correlation matrix between two spaces^37^. To favor interpretable latent components such that each component is associated with a small subset of the task variables, we imposed a sparsification penalty (least absolute shrinkage and selection operator with fullness constant of 0.3 on the weights of the task variables^37^). This regularization helps reduce overfitting the model. We calculated ten components for each training set (Fig. 4b), and then identified neural components making the greatest contributions to rewards, choices, waiting time and reward ratio. Interestingly, the waiting time neural component ramps up between the consecutive presses (Fig. 4b, third row), suggesting that the latent representation of the waiting time might be used by the brain to generate the next press, in a similar fashion to the evidence accumulation models proposed in decision making^38^. The reward ratio component followed the difference between the reward ratio of the boxes (Fig. 4b, fourth row). The reward and choice components showed sharp post-press elevated activity (Fig. 4b, first and second rows).

**Extended Data Fig. 6:**
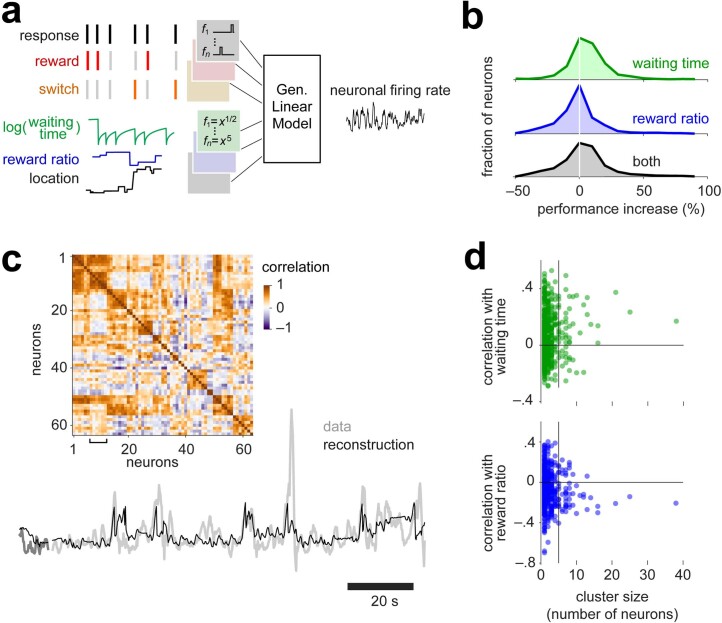
An encoding model for reconstructing the activity of individual neurons using the continuously evolving experimental variables. **(a)** A generalized linear model to reconstruct continuously evolving firing rates of individually recorded neurons. Rates are predicted from combinations of the task variables passed through a set of basis functions. The basis functions were pulse-shaped temporal delay filters for press, reward, and choice events. (The time of the reward and the choice events were assumed to match the time of the press after which the reward was delivered or the choice was made.) The basis functions for continuously evolving task variables (waiting time, reward ratio, and 2-dimensional location) were instantaneous power functions with powers of ½, 1, 2, 3, and 5. Altogether, 51 predictors were made using these 6 task variables. The model was fit to the training data using a Gaussian likelihood function; the trained model was used to reconstruct the neural activity for held-out testing data. **(b)** The improvement in the performance of the model when either one or both reward predictors were used alongside the other task variables in (a). Improvement was calculated as the percentage increase in the correlation between the recorded and reconstructed activities. While the waiting time improved the model performance for the entire population by 5% ($p$≪${10}^{-3}$), this improvement was insignificant for the reward ratio (*p* = 1) at the level of individually recorded neurons. **(c)** The reconstructed and recorded firing rates averaged across 6 neurons in a sample session. The neurons were selected from 60 simultaneously recorded neurons in this session by clustering them using the correlation matrix of their reconstructed activities (inset), then choosing all neurons in a sample cluster (bracket in the inset) to show here. **(d)** The correlation coefficient between each reward predictor and the reconstructed neural activity averaged across neurons in the same cluster *vs*. the number of neurons in the cluster. The vertical line shows a cluster size of 5, which was the lower bound of the cluster size for the clusters that were included in Fig. 4c. Across sessions, the average firing rates of clusters of ≥ 5 neurons were positively correlated with the waiting time (*p* = 0.004, *top*) and negatively correlated with the reward ratio (*p* = 0.004, *bottom*).

**Extended Data Fig. 7:**
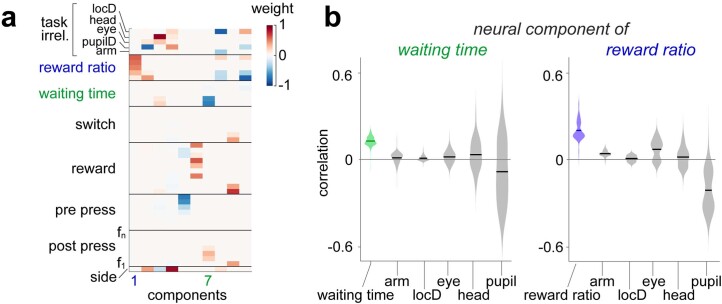
Extending the canonical correlation analysis from Fig. 4 to include eye and limb movements of monkey M. **(a)** The weight of the contribution of each task variable in the 10 first canonical components, sorted in descending order of the components’ correlations between the task and neural spaces. The indices of the components representing waiting time and reward ratio are color coded. **b)** *Left*: Pearson correlation coefficient between component 7, the neural component, best associated with the waiting time, and the true waiting time, as well as between this component and any of the task-irrelevant variables. *Right*: same as left but for neural component 1 instead of component 7, and reward ratio instead of waiting time. The mean (lines) and the Gaussian-smoothed distribution (shades), pooled across held-out chunks of all sessions are shown.

We asked whether fitting a model to reconstruct the activity of individually recorded neurons^34^ or sites^33^, then clustering the neurons based on the similarity between the reconstructed activity (Extended Data Fig. 6) yields a better representation than the latent variables that we found using the CCA. We calculated the Pearson correlation coefficient between reward predictors and their associated canonical components analysis and compared them with the correlation between the reward predictors with the neuronal clusters or individual neurons in each session that was maximally correlated with each reward predictor. The average correlation coefficient between the waiting time and the neural components was higher than that with the individual neurons or neural clusters of >5 neurons (WRFDR, *P* < 0.005; Fig. 4c, left). The correlation between the reward ratio and the neural components was the same as that with the individual neurons or clusters (WRFDR, *P* > 0.1; Fig. 4c, right). This indicates that the latent neural components provide better correlates of reward predictors relative to individual neurons or the average activity of groups of neurons that were clustered together based on their task-relevant activity. Furthermore, the latent neural components were uncontaminated by movement-related confounds (Extended Data Fig. 7).

### Predicting reward, choice and the next waiting time

Since the animal cannot know the true hidden reward dynamics, its choices can only be driven by its subjective beliefs about these variables, rather than the objective truth from the experiment. For instance, if the monkey overestimates reward probability (perhaps due to misjudging waiting time or scheduled reward rate), he is more likely to switch boxes after an unrewarded push. We predicted switching based on neuronal components corresponding to task variables, interpreting them as current estimates of the animal’s subjective beliefs. We decoded the pre-press neural activity by projecting the population activity onto the subspace formed by the first ten canonical components for the reward predictors. This projection accounts for latent representation of reward predictors that could potentially influence the choices or the next waiting time or predict the eventual reward outcome.

**Fig. 5:**
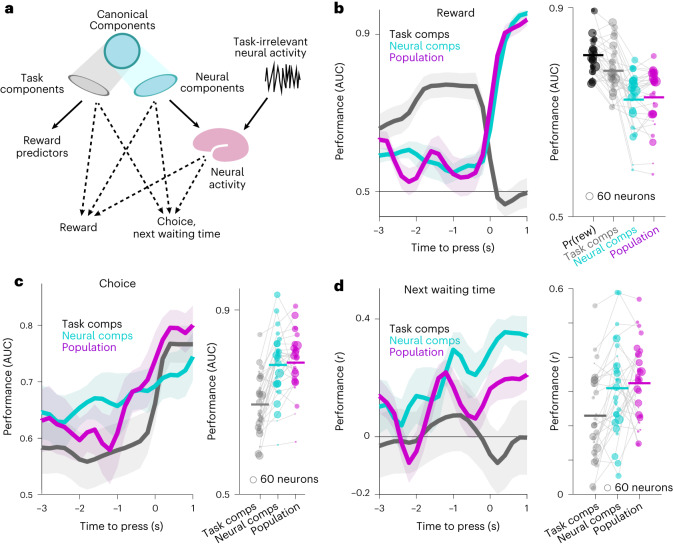
Neural population analysis. **a**, Prediction of rewards, choice or next waiting time from task components, neural components and the entire simultaneously recorded population. The task components are defined as the projection of the canonical component in the ten-dimensional space of waiting time and reward rate (each passed through five basis functions). The neural components are defined as the projection of the canonical component into the neural population space. The prediction was trained and tested for each 200 ms time bin, starting 3 s before and ending 1 s after the presses. **b**–**d**, The prediction results for rewards (**b**), choice (**c**) or next waiting time (**d**) for example sessions (left) or summarized across all sessions for the peak within the 2 s time interval before the presses (right). Left: the prediction performances using the post-press components or population activity are shown for comparison. Data are presented as mean values ± s.e.m. Right: the peak was calculated as the average of five time bins with the highest prediction performances. The prediction performance for the pre-press time bins provide evidence that the post-press choices or the rewards were in fact predictable, even before the presses were made.

We attempted to predict rewards, choices and the next waiting times from three distinct types of predictors: (1) the pre-press reward predictors (canonical components in the reward predictors’ space), (2) neural representations of the reward predictors (canonical components in the neural space) and (3) the entire simultaneously recorded neural population (Fig. 5a). For a fair comparison between the components and the entire neural population, we did not sparsify the weights of task variables in canonical components. To predict the reward, we trained binomial logistic regressions on the same data used to find the canonical components, then tested on the held-out data. To assess the prediction performance, we calculated the AUC showing the discriminability of the predictors’ output for the rewarded presses from the unrewarded presses. The same method was used for the choice to stay or switch. To predict the next waiting time, we used generalized linear models instead of logistic regression and evaluated the performance by calculating the Pearson correlation coefficient between the real and the predicted values. All predictors were trained and tested for each 200 ms time bin, starting 3 s before each press and ending 1 s after.

**Extended Data Fig. 8:**
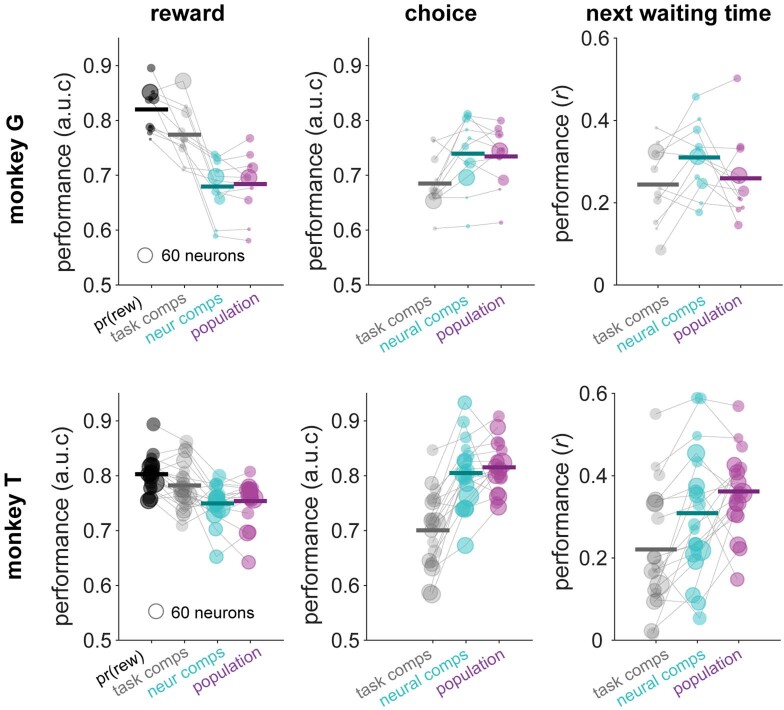
Prediction of rewards, choice, or next waiting time from task components, neural components, and the entire simultaneously recorded population. same as Fig. 5b–d, but separated for monkeys G and T.

In the example session shown in Fig. 5b, left, the prediction of the reward outcomes using the task components improved as the analysis windows approached the time of the press. The reward outcomes are determined by the actual experimental task variables, and indeed we confirmed that the true pre-press task components (the projection of the canonical component in the space of reward predictors) predict actual rewards better than either their neural representations (the projection of the canonical components on the neural population space) or the entire neural population (Fig. 5b, right, and for monkey-separated results, see Extended Data Fig. 8).

**Extended Data Fig. 9:**
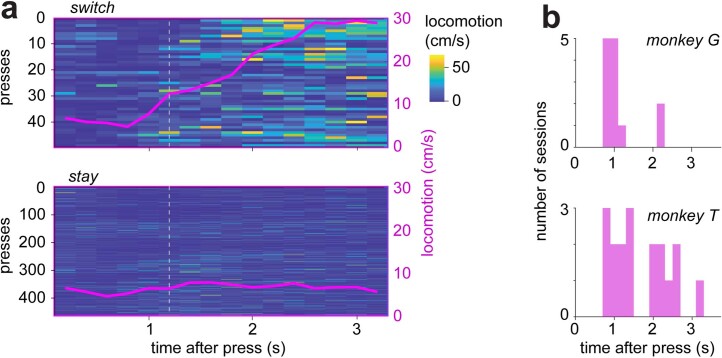
Detecting the time of the switches using the locomotion data. **(a)** The magnitude of locomotion in each 200 ms time bin, separated for switch and stay trials. Trial averages are shown as solid yellow lines. The white dashed line shows when the average locomotion speed in switch trials is greater than 3 SD of the magnitude of locomotion in stay trials. **(b)** Average switch time in each session.

In contrast, the choices and the next waiting time should follow the animal’s subjective estimation of the reward dynamic variables. Fascinatingly, the neural activity before a press predicted the subsequent choice (Fig. 5c) and waiting time (Fig. 5d). As the animal’s movement to switch to the other box or press the button again occurred after the current press (Extended Data Fig. 9), the prediction of either of these actions by the neural components precedes the execution of the predicted actions. Moreover, the head, arm and eye movements within the pre-press time window were not significantly different between presses after which the animal stayed and the presses after which the animal switched to the other box (Extended Data Fig. 5c; *P* > 0.12 for all the comparisons, Wilcoxon rank-sum test). Therefore, we provide further evidence that the animals construct an expectation of reward before a press, based on their subjective understanding of the temporal structure of the task. Subsequently, animals decide when and where to press next based on the expected reward and the actually observed reward. Interestingly, the ten-dimensional neural representation of the pre-press task components predicted the choice and the next waiting time as well as the entire neural population (Fig. 5c,d), indicating that these few canonical neural components successfully capture the relevant signals within the larger neural population space.

It might seem obvious that neural features should be better predictors of when and where to press than experimental variables, after all, the animal’s brain is making its choice and not the experimental equipment. However, it is not evident a priori that the relevant neural representations would be found within our recorded dlPFC population, nor whether we record enough neurons to capture enough of the animal’s choice-relevant information. Furthermore, even if the dlPFC does contain the choice-relevant signals, it is not obvious that the neural components for our specific hypothesized reward predictors would be the right ones to predict the choices. It is thus noteworthy that these neurally decoded reward predictors predict choices significantly better than the task variables from which they are derived, and equally well as the full neural population. Evidently, our analysis identifies a neural subspace containing correlates of latent variables that are relevant for subsequent choices. This subspace also tends to avoid neural dimensions that contain choice-irrelevant variability, since if present, these variations could contribute to overfitting and would only hinder our ability to predict choice. We conclude that we are capturing neural correlates of the animals’ subjective beliefs about the latent reward dynamics that inform their choices.

## Discussion

We used a trial-free, unrestrained-animal approach to demonstrate that freely moving monkeys base their foraging strategy on an internal prediction of reward. This prediction is not based solely on the recent history of reward but relies on an internal estimation of the time they have been waiting since the last time they made a choice, which determines the probability of reward availability. Indeed, we found that neural populations in the prefrontal cortex contain information about reward predictors. Complementary to previous research in restrained animals^8,13^, we revealed that neural signals not only encode reward information, but also significantly predict animal’s choices after each press during foraging. These findings challenge and extend long-standing theories of reward-seeking behavior^9^ that suggest that animals follow the choice with the maximum recent rate of reward, without constructing a reward model to predict future behavior, according to the matching law^9^.

**Extended Data Fig. 10:**
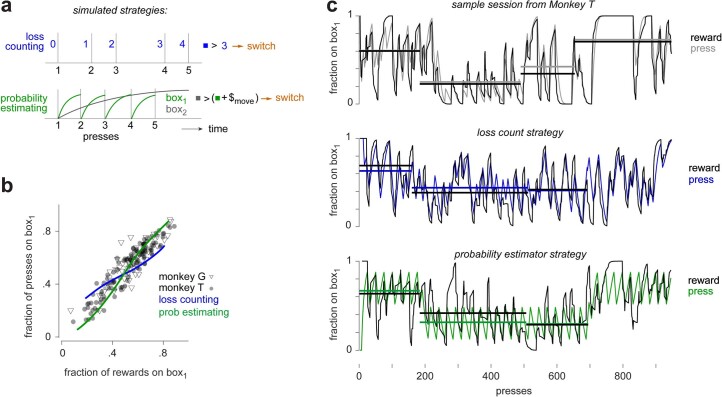
Analysis of the matching behavior for monkeys G and T and two simulated agents. **(a)** Illustration of foraging strategies for two simulated agents. The ‘*loss counting’* agent switches to the other box when the *loss count* exceeds a threshold drawn from a Gaussian distribution (λ = 2.66, σ = 1.9). The ‘probability estimator’ agent switches to the other box when the probability of reward availability on the other box exceeds the probability of reward availability in the current box by a fixed switching cost^1^. The inter-press times were drawn from a random geometric distribution for both agents. The parameters of these strategies, namely the *loss count* distribution, the inter-press time distribution, and the switching time were estimated from the behavior of the monkeys. Each agent was simulated for 100 rewards for each set of reward schedules for box_1_ and box_2_. The variable interval reward schedules spanned the range between VI-5 and VI-50 in steps of 1 s and were drawn independently for each box. **(b)** Matching behavior of two monkeys and two simulated agents: the fraction of behavioral presses at box 1 is approximately proportional to the fraction of rewards at box 1 (26 sets of schedules for monkey G and 59 sets of schedules for monkey T are shown). Curves show spline fits for the matching behavior for the simulated agents. **c)** Dynamic matching for a sample session of Monkey T with 3 sets of reward schedules: VI15-VI25, VI25-VI15, and VI15-VI25 again. We compared two simulated agents of panel a. The reward and press rates were calculated locally using a causal half-Gaussian filter^8^.

We argue that matching, while ubiquitous, does not entail a single computational strategy. For our foraging task, matching behavior is consistent with substantially different strategies. One strategy can be simulated using an agent that switches to the other box after the number of unrewarded presses exceeds a noisy threshold (Extended Data Fig. 10). This strategy corresponds to a basic ‘win-stay/lose-switch’ rule. We implemented this strategy by sampling the threshold from a Gaussian distribution with the same mean and variance as the loss count distribution at times when the animal switches sides. Although this agent is blind to both the average reward rate and the probability of the next reward, it still follows the generalized matching law (Extended Data Fig. 10). The slight undermatching that we observed resembles the behavior of various species in previous studies^7,9^.

We examined a more complex strategy that tracks reward probability and uses foraging theory to make choices by involving three variables: (1) the time since the preceding press and (2) the variable-interval schedule—which together determine the probability of reward—and (3) the relative cost of switching locations, which affects the threshold for when to switch. We simulated an agent that follows such a strategy by making choices based on the correct probability of reward availability on both boxes. The agent switches to the other side when the probability of reward availability on the other box exceeds that of the current box by a fixed switching cost, and otherwise waits for the probability of reward availability to increase everywhere (Extended Data Fig. 10), in accordance with the marginal value theorem of foraging theory^1^. Unlike the first agent, this agent has complete information about the task. Nonetheless, we again observed nearly matching behavior, now with slight overmatching (Extended Data Fig. 10). These two simulations show that the generalized matching law may arise when following a strategy that is either blind to timing or fully informed. This implies that matching behavior is not, by itself, informative about the underlying strategy or animals’ ability to grasp the hidden rule of the task.

Surprisingly, we found that the targeted representation within the high-dimensional space of neural population activity predicts choice better than the behavioral dynamics and as well as the entire population of recorded neural activity. This is an important confirmation of how targeted dimensionality reduction can reveal neural computations better than behavior or unprocessed neural activity. This type of analysis is essential in natural experiments where task variables are correlated.

One limitation of our findings is the extent to which our results can be generalized across other types of reward dynamics. The reward dynamics in our task are stochastic and time based, and they resemble the repletion of food resources found in nature. Follow-up studies are needed to determine whether our findings apply to other reward schemes, such as non-Markovian, more clock-like dynamics or those based on press rate^39^, whereby the reward becomes available after a variable number of presses rather than a variable time interval.

By allowing animals to move freely, our study represents a necessary move toward studying neural correlates of natural cognition in a free-roaming setting. This paradigm shift has been suggested decades ago^40^, but is only feasible now due to advances in low-power, high-throughput electrophysiological devices and large-scale computing^41^. Freely moving experimental paradigms probably increase the engagement of natural decision-making processes in the animal’s brain, and possibly reduce the distortions in population dynamics that may be associated with unnatural head-fixed tasks. The free-roaming setting also enabled us to implement a natural switching cost between two reward options by simply allowing the monkey to walk between them. This is commonly implemented for restrained animals as a timeout period immediately after switching decisions. The subjective value and its neural representation potentially differ for a foraging task in which the animal explores by performing an effortful action such as relocation, compared twith a task in which explorative actions do not cost effort, but instead cost time. Overall, a shift toward more natural behavior will be inevitable for understanding neural mechanisms of cognition^41–46^.

## Methods

All experiments were performed under protocols approved by the University of Texas at Houston Animal Care and Use Committee and the Institutional Animal Care and Use Committee for the University of Texas Health Science Center at Houston. Two adult male rhesus monkeys (*Macaca* *mulatta*; monkey G: 15 kg, 9 years old; monkey T: 12 kg, 9 years old) were used in the experiments. An additional adult male rhesus monkey (*Macaca* *mulatta*; monkey M, 10 kg, 11 years old) was used for the control experiment, tracking the eye and limb movements.

### Behavioral training and testing

The experimental setup was a custom-made cage (120 cm × 60 cm × 90 cm) that was placed in a dedicated room, free from distractions. After habituating each monkey for at least 4 days per week for over 4 weeks, we trained animals to press the button on each box to receive a reward. Over the course of 4–6 months, we gradually increased the mean time in the VI schedule to let the monkeys grasp the concept of probabilistic reward delivery. Once we started using VI 10 (corresponding to an average reward rate of <0.1 rewards s^−1^), monkeys started to spontaneously switch back and forth between the two boxes. If the monkeys disengaged from the task or showed signs of stress, we decreased the VI schedule (increased the reward rate) and kept it constant for 1 or 2 days. If the monkey showed a strong bias toward one reward source, we used unbalanced schedules to encourage the monkeys to explore the less preferred box.

After training, we tested monkeys using a range of balanced and unbalanced reward schedules. For balanced schedules we used VI reward scheduling with the average interreward interval of 20 s or 30 s, that is, VI 20 or VI 30, on both boxes. For unbalanced schedules, we used VI 20 versus VI 40, VI 15 versus VI 25, or VI 10 versus VI30. The unbalanced schedules may reverse once, twice or three times during a session, for example, after a reversal the box with VI 20 becomes VI 40 and the box with VI 40 becomes VI 20. Each session lasts until the monkey receives 100 or 200 rewards, ranging from 1 to 7 h including a 1 h break after 100 rewards in sessions with 200 rewards. If monkeys were not engaged with the task for more than 2 min, we sometimes interrupted them to encourage them to engage with the task. For the analysis, we exclude all presses that occurred for more than 60 s. For the press-locked analysis, we also excluded presses that were made less than 2 s after the previous press to avoid mixing in the press-locked neural activity.

### Tracking whole-body, limb and eye movements

To determine the physical location and locomotion of the monkey, an overhead wide-angle camera was permanently installed in the experimental cage and the video was recorded at an average rate of six frames per second. Each frame was postprocessed in six steps using custom-made MATLAB code. First, the background image was extracted by averaging all frames in the same experimental session, then it was subtracted from each frame. The background-subtracted image was then passed through a manually determined threshold to identify the dark areas. The same image frame was also processed using standard edge detection algorithms. The thresholded and edge detected images were then multiplied together, and the result was convolved with a spatial filter, which was a circle with the estimated angular diameter of the monkey. The peak of this filtered image was marked as the location of the monkey. We used this heuristic because the illumination of the experimental room and the configuration of objects was constant. We expect novel techniques for motion and posture detection using deep neural network^47,48^ to yield similar results. Locomotion (speed) was calculated as the magnitude of the vector difference between monkey locations in consecutive frames divided by their time difference.

To compute the head, arm and whole-body movements of monkey M, we trained DeepLabCut (version 2.0) with 200 frames annotated with the center of the head, snout, each ear, shoulders, elbows and paws, then tracked these body markers in videos recorded in three sessions. These frames came from the same overhead camera described above but were recorded at 30 Hz. ‘Head movements’ included labels from the center of the head, snout and each ear. ‘Limb or arm’ movement was computed from shoulder, elbow and paw labels. ‘Torso or whole-body’ movement was calculated from the animal’s upper and mid back labels. To calculate the average speed of each label during frames of interest, we calculated the Euclidean distance between the label coordinates across consecutive frames divided by the time between frames. Subsequently, we quantified the overall movement of each body area (head, arm or torso) by computing the mean speed, and averaging the speeds of the corresponding body part labels that were recorded at 30 Hz.

To track the eyes, we used a commercially available eye tracker (ISCAN). To train animals to wear the device without damaging it, its three-dimensional (3D) geometry was modeled (Sketchup Pro), and dummies were 3D printed and fitted with eye mirrors. To properly position the eye tracker and dummies relative to the eye, custom adapters were designed and 3D printed to attach directly to the animal’s head post and serve as an anchor point for the eye tracker. These adapters were designed to interface with the head post, without touching the animal directly, to minimize discomfort and reduce the likelihood of the device being tampered with. These dummy eye trackers were worn by animals for several mock recording sessions to adjust them to wearing the device. Once the animals grew accustomed to wearing the dummy, the real device was used. We used two-dimensional coordinates of the pupil to compute eye velocity from Euclidean distance of these values across consecutive eye camera frames.

### Determining reward availability and calculating probability of reward availability

In each time bin of size d*t* = 10 ms, reward became available at a given box if a sample from a Bernoulli distribution was 1. The probability of this event was *dt*/VI. When the reward became available, it stayed available until collected by the animal. This makes the probability of reward availability a function of the scheduled VI as well as the time since the preceding press:

$${P}_{\mathrm{rew}}=1-{\left(1-{{\mathrm{d}}t}/\mathrm{VI}\right)}^{t/{{\mathrm{d}}t}},$$

where *t* is the time since the preceding press (Extended Data Fig. 1).

### Chronic implantation of the Utah array

A titanium head post (Christ Instruments) was implanted, followed by a recovery period (>6 weeks). After acclimatization with the experimental setup, each animal was surgically implanted with a 96-channel Utah array (BlackRock Microsystems) in the dlPFC (area 46; anterior of the Arcuate sulcus and dorsal of the principal sulcus (Extended Data Fig. 1)). The stereotaxic location of dlPFC was determined using magnetic resonance images and brain atlases before the surgical procedure. The array was implanted using the pneumatic inserter (Blackrock Microsystems). The pedestal was implanted on the caudal skull using either bone cement or bone screws and dental acrylic. Two reference wires were passed through the craniotomy under and above the dura mater. After the implant, the electrical contacts on the pedestal were protected always using a plastic cap except during the experiment. Following array implantation, animals had at least a 2 week recovery period before we recorded from the array.

### Recording and preprocessing of neural activity

To record the activity of neurons while minimizing the interference with the behavioral task, we used a lightweight, battery-powered device (Cereplex-W, Blackrock Microsystems) that communicates wirelessly with a central amplifier and digital processor (Cerebus neural signal processor, Blackrock Microsystems). First, the monkey was head fixed, the protective cap of the array’s pedestal was removed, the contacts were cleaned using alcohol and the wireless transmitter was screwed to the pedestal. The neural activity was recorded in the head fixed position for 10 min to ensure the quality of the signal before releasing the monkey in the experimental cage. The cage was surrounded by eight antennas. In the recorded signal, spikes were detected online (Cerebus neural signal processor, Blackrock Microsystems) using a manually selected upper threshold on the amplitude of the recorded signal in each channel or an upper and a lower threshold that were ±6.25 times the standard deviation of the raw signal. To minimize the recording noise, we optimized the electrical grounding by keeping the connection of the pedestal to the bone clean and tight. The on-site digitization in the wireless device also showed lower noise than common wired head stages. The remaining noise from the movements and muscle activities of the monkeys was removed offline using the automatic algorithms in offline sorting (Plexon Inc.). Briefly, this was done by removing the outliers (outlier threshold, 4–5 standard deviations) in a 3D space that was formed by the first three principal components of the spike waveforms. Then, the principal components were used to sort single units using the expectation-maximization algorithm (offline sorter version 4.0). Each single and multi-unit signal was evaluated using several criteria: consistent spike waveforms, modulation of activity with 1 s of the button pushes and exponentially decaying inter-spike interval histogram with no inter-spike interval shorter than the refractory period (1 ms). The analyses used all spiking units with consistent waveform shapes (single units) as well as spiking units with mixed waveform shapes but clear pre- or post-press modulation of firing rates (multi-units).

### Removing task-irrelevant components from neural activity

For each neuron *k*, we remove movement-related temporal components of the press *r*_*kt*_, by subtracting its projection onto the subspace spanned by the task-irrelevant variables: ${r}_{k}^{\perp }={r}_{k}-\varPi {r}_{k}$, where *Π* is the projection matrix $\varPi =L{\left(L{L}^{\top }\right)}^{-1}{L}^{\top }$ and *L* is the *T* × 1 vector describing the time series of locomotion, calculated as the magnitude of the changes in the two-dimensional location.

### Regression-based and binary decoder analysis

To decode a binary variable, such as the reward or the choice to stay or switch, we used logistic regression. To evaluate this model, we used the AUC to determine the separability of the probability distributions of the held-out samples belonging to either of the classes (reward versus no reward and stay versus switch). To decode continuous-value variables such as waiting time or the reward ratio, we used a linear regression model^49^. To evaluate this model, we calculated the Pearson correlation coefficient between the measured and predicted values. To train and cross-validate these decoders, we divided the presses in each session to 4–18 blocks, holding out one block at a time for testing and using the rest of the blocks for training. To divide the presses into blocks, we found the gaps in press times that were larger than 30 s, then placed all presses between consecutive gaps in one block.

### Selecting task-relevant variables and useful basis functions for continuous-time analyses

Our continuous-time analyses used a set of basis functions applied to the time series of experimental task variables. To use event-based variables in continuous-time predictions, we filtered the variables with different boxcar-shaped delay filters. We used different numbers of these pulse basis functions for different variables: seven basis functions for the pre-press time interval, post-press time and post-choice; and ten basis functions for the post-reward time to include the entire range of reward collection time, starting with the food release sound cue at the press time and ending when the food pellet was consumed (spanning 2 s; Fig. 4a). For task variables *x*_*t*_ that were already defined continuously over time, we applied a set of nonlinear power functions, ${x}_{t}^{a}$, with powers $a\in \left\{\tfrac{1}{2},1,2,3,5\right\}$. This 51-dimensional feature vector was used to predict components of neural activity.

### CCA

Canonical components were calculated using singular value decomposition of the cross-covariance matrix between the task variables and the neural activity, specifically the pre-press firing rates of simultaneously recorded neurons. We regularized this linear model using an *ℓ*_1_ penalty^37^ to calculate canonical components. The cross-validation procedure was the same as for the decoders.

### Statistical analysis

We used the two-sided Wilcoxon signed-rank test except where indicated. We chose this test rather than parametric tests, such as the *t*-test, for its greater statistical power (lower type I and type II errors) when data are not normally distributed. When multiple groups of data were tested, we used the false discovery rate multiple comparisons^50^ correction whose implementation is a standard function in MATLAB. We used WRFDR abbreviation to indicate using two-sided signed-rank test with false discovery rate multiple comparison correction. No statistical methods were used to predetermine sample sizes. However, the size of our dataset and the number of the experimental sessions are similar to those reported previously^27^. Data collection and analysis were not performed blind to the conditions of the experiments.

### Use of generative artificial intelligence

While preparing the last version of this work, the authors used chatGTP to shorten parts of the text to meet the journal’s word count limit. After using this tool, the authors reviewed and edited the content as needed. The authors take full responsibility for the content of the publication.

### Reporting summary

Further information on research design is available in the Nature Portfolio Reporting Summary linked to this article.

## Online content

Any methods, additional references, Nature Portfolio reporting summaries, source data, extended data, supplementary information, acknowledgements, peer review information; details of author contributions and competing interests; and statements of data and code availability are available at 10.1038/s41593-024-01575-w.

## Supplementary information

Reporting Summary

## Data Availability

The preprocessed data used for this study is available at 10.6084/m9.figshare.24762996.v1. The raw data will be available upon request.

## Code availability

The custom code used in this study is available at 10.6084/m9.figshare.24762996.v1.
